# Polyoxometalate steric hindrance driven chirality-selective separation of subnanometer carbon nanotubes†

**DOI:** 10.1039/d2sc01160c

**Published:** 2022-04-25

**Authors:** Xusheng Yang, Chao Zhu, Lianduan Zeng, Weiyang Xue, Luyao Zhang, Lei Zhang, Kaitong Zhao, Min Lyu, Lei Wang, Yuan-Zhu Zhang, Xiao Wang, Yan Li, Feng Yang

**Affiliations:** Department of Chemistry, Guangdong Provincial Key Laboratory of Catalysis, Southern University of Science and Technology Shenzhen Guangdong 518055 China yangf3@sustech.edu.cn; Shenzhen Key Laboratory of Nanobiomechanics, Shenzhen Institutes of Advanced Technology, Chinese Academy of Sciences Shenzhen 518055 China xiao.wang@siat.ac.cn; Beijing National Laboratory for Molecular Science, Key Laboratory for the Physics and Chemistry of Nanodevices, State Key Laboratory of Rare Earth Materials Chemistry and Applications, College of Chemistry and Molecular Engineering, Peking University Beijing 100871 China yanli@pku.edu.cn; Peking University Shenzhen Institute Shenzhen 518057 China; PKU-HKUST ShenZhen-HongKong Institution Shenzhen 518057 China; Nano Science and Technology Institute, University of Science and Technology of China Suzhou 215000 China

## Abstract

Subnanometer single-chirality single-walled carbon nanotubes (SWCNTs) are of particular interest in multiple applications. Inspired by the interdisciplinary combination of redox active polyoxometalates and SWCNTs, here we report a cluster steric hindrance strategy by assembling polyoxometalates on the outer surface of subnanometer SWCNTs *via* electron transfer and demonstrate the selective separation of monochiral (6,5) SWCNTs with a diameter of 0.75 nm by a commercially available conjugated polymer. The combined use of DFT calculations, TEM, and XPS unveils the mechanism that selective separation is associated with tube diameter-dependent interactions between the tube and clusters. Sonication drives the preferential detachment of polyoxometalate clusters from small-diameter (6,5) SWCNTs, attributable to weak tube–cluster interactions, which enables the polymer wrapping and separation of the released SWCNTs, while strong binding clusters with large-diameter SWCNTs provide steric hindrance and block the polymer wrapping. The polyoxometalate-assisted modulation, which can be rationally customized, provides a universal and robust pathway for the separation of SWCNTs.

## Abstract 2

We develop a cluster steric hindrance strategy by assembling polyoxometalates on subnanometer single-walled carbon nanotubes (SWCNTs) and demonstrate the selective separation of single-chirality (6,5) SWCNTs *via* polymer extraction.

## Graphical abstract

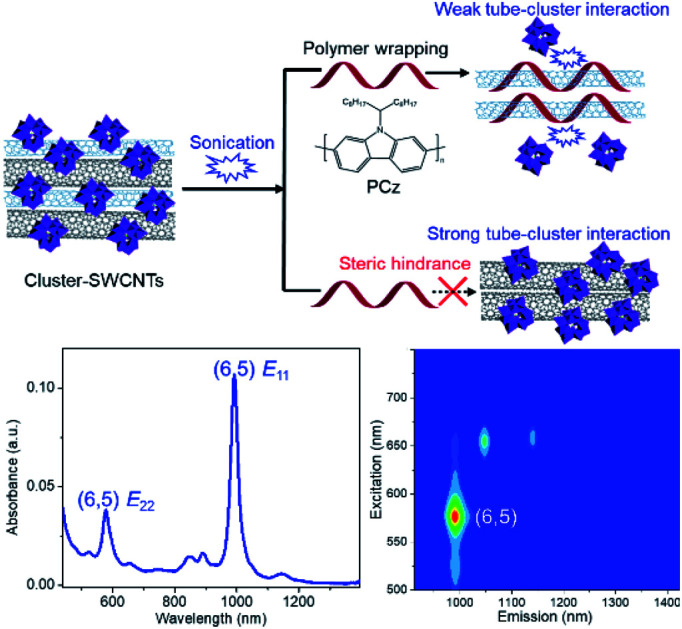

## Introduction

Single-walled carbon nanotubes (SWCNTs)^1^ are of tremendous interest in research because of their unique structure/chirality (*n*,*m*) dependent properties and their diverse applications, in particular for electronics,^2–5^ photonics,^6–9^ and bioimaging.^10^ Subnanometer (<1 nm) SWCNTs with large bandgaps are ideal for solar cell applications,^11^ and they could be beneficial as part of the active absorbing layer in solar cells because their wider bandgap would allow the formation of an improved heterojunction.^12^ The structure controlled synthesis of SWCNTs has been developed over nearly three decades,^13–25^ in which subnanometer SWCNTs with controlled structures are of immense interest.^26–28^

There has been significant progress in the solution processing based sorting of SWCNTs with controlled structures, including ultracentrifugation,^29,30^ electrophoretic^31^ and/or chromatographic separation,^32–35^ aqueous two-phase separation,^36–41^*etc.* Among them, the one-pot separation of semiconducting SWCNTs by conjugated polymers is of particular interest and has become the method of choice for circuit and solar cell applications due to the simplicity, high purity, and high thin film quality.^42–49^ The separation of single-chirality SWCNTs using conjugated polymer extraction is one of the long-standing goals. To date, the sorting of chirality enriched SWCNTs has been achieved using several types of polymers.^50–55^ Most recently, Li *et al.* demonstrated the semiconducting separation of conjugated polymer-wrapped SWCNTs larger than 1 nm, followed by ultracentrifugation and stepwise extraction processing to yield monochiral (10,8) and (12,5) SWCNTs with diameters larger than 1 nm.^56^ The discovery of a one-pot sorting strategy using a conjugated polymer extraction technique with specific chirality selectivity is a critical issue in the current field.

The key issue for the selective separation of SWCNTs is to amplify the differences (*e.g.*, density, tube length, redox property) between various SWCNT species in a polydisperse mixture.^57–60^ Conjugated polymers are generally considered to disperse SWCNTs by wrapping around nanotubes through noncovalent π–π interactions. Polymer wrapping would be inhibited if there was a steric hindrance on outside SWCNTs. Polyoxometalates, which possess enormously versatile structures and compositions,^61,62^ are considered to be ideal electron acceptors when electrostatically bonding with electron donors such as SWCNTs.^63–69^ For example, Newton *et al.* recently reported that polyoxometalates are spontaneously encapsulated by SWCNTs, driven by a redox reaction between the polyoxometalates and the SWCNTs. These redox materials exhibit exceptional electrochemical stability.^70–72^ We demonstrated that the formation of electron donor–acceptor systems composed of SWCNTs and encapsulated polyoxometalates is effective to modulate the interaction of inter-tubes, thus selectively extracting large-diameter semiconducting SWCNTs.^73,74^ The adaptability in chemical synthesis provides a wide variety of customized polyoxometalates with tunable redox potentials, which enables precise modulation of the electronic properties of SWCNTs.

**Fig. 1:**
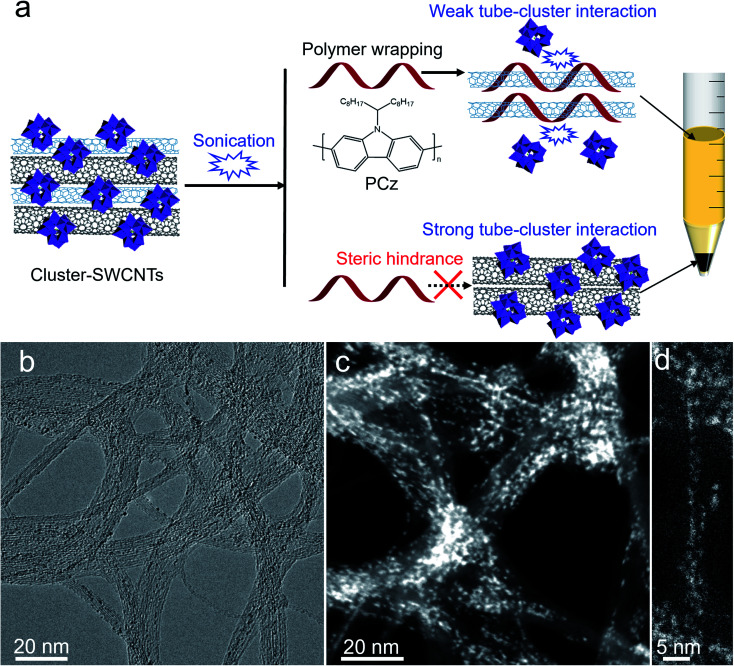
(a) Schematic diagrams of the selective separation of subnanometer SWCNTs through the steric hindrance effect of polyoxometalate clusters. (b) TEM and (c and d) HAADF-STEM images of {PW_12_} on the outside of subnanometer SWCNTs.

Herein, inspired by the interaction between SWCNTs and polyoxometalates, we chose polyoxometalate clusters as steric hindrance to adsorb on the outer surface of subnanometer SWCNTs. During sonication, the clusters preferentially detached from certain SWCNTs with weak interactions, thereby enabling the polymer to selectively wrap and separate SWCNTs, while the strong binding of other SWCNT bundles with the clusters blocked the polymer wrapping (Fig. 1a). With this strategy, the separation of subnanometer (6,5) SWCNTs was achieved, which was characterized with ultraviolet-visible-near infrared (UV-vis-NIR) absorption and photoluminescence (PL) spectroscopies. The sorting mechanisms associated with the tube diameter-dependent interaction between SWCNTs and polyoxometalates were unveiled by density functional theory (DFT) calculations, spectroscopy, transmission electron microscopy (TEM), and X-ray photoelectron spectroscopy (XPS). This sorting strategy was further proven to be valid in a series of polyoxometalates with appropriate oxidizing abilities.

## Results and discussion

### Assembly of polyoxometalate clusters on subnanometer SWCNTs

We used the commercialized CoMoCAT SWCNTs (CG100, CO deposition on Co–Mo catalyst, denoted as CoMo-SWCNTs), the smallest-diameter mass produced SWCNTs with a diameter range of 0.7–0.9 nm, to assemble with the Keggin type polyoxometalate H_3_PW_12_O_40_ (denoted as {PW_12_}-SWCNTs) (see the Experimental section). Fig. 1b–d and S1† depict the TEM and high-angle annular dark field scanning TEM (HAADF-STEM) images of cluster–SWCNT hybrids. Most of the ∼1 nm {PW_12_} clusters are on the outside of the subnanometer CoMo-SWCNTs due to the limited size of the tube cavity (<1 nm). The loading of {PW_12_} clusters on the SWCNTs was determined to be 27 wt% based on the thermogravimetric analysis (Fig. S2†). We did not observe a notable color change to dark blue after adding subnanometer CoMo-SWCNTs into {PW_12_} aqueous solution, which is different from the phenomenon observed in previous reports when encapsulating {PW_12_} into large-diameter (1–2 nm) SWCNTs to form heteropoly blues.^70,73^ It was further found that there was no obvious shift for the *G* band of {PW_12_}-out-SWCNTs, compared with that of pristine SWCNTs (Fig. S3†). This phenomenon is also different from previous reports that there exists an up-shift of the *G* band when {PW_12_} is encapsulated in large-diameter (1–2 nm) SWCNTs,^63,70,72^ indicating the oxidation of the SWCNTs. The possible reason is that the degree of {PW_12_} reduction in {PW_12_}-out-SWCNTs is smaller than that in {PW_12_}-in-SWCNTs, that is, the weaker interaction of {PW_12_}-out-SWCNTs than {PW_12_}-in-SWCNTs. Despite the differences, electrons were also found to transfer from the SWCNTs to the outer surface of {PW_12_}, revealed by XPS (Fig. S4†), affirming that there exists weak interactions within the {PW_12_}-SWCNTs. Due to the weak interactions between the polyoxometalates and subnanometer SWCNTs, it is possible to achieve the sonication triggered detachment of clusters from the SWCNTs.

### Selective separation of the SWCNTs

In order to check the chirality distribution of the raw CoMo-SWCNTs, the non-selective sodium deoxycholate was used to disperse the raw CoMo-SWCNTs, which shows that the raw CoMo-SWCNTs do not exhibit obvious chirality selectivity (Fig. S5†). The commercially available linear homopolymer poly[9-(1-octylonoyl)-9*H*-carbazole-2,7-diyl] (denoted as PCz)^46^ was used to disperse and separate the SWCNTs. 5 mg of {PW_12_}-SWCNTs and 10 mg of PCz were mixed in 20 mL of toluene. The resulting mixture was sonicated by tip sonication under 500 W for 150 min and then the suspension was centrifuged to separate the well-dispersed SWCNTs and remove the aggregates of the tube bundles and clusters wrapped by the polymer.

**Fig. 2:**
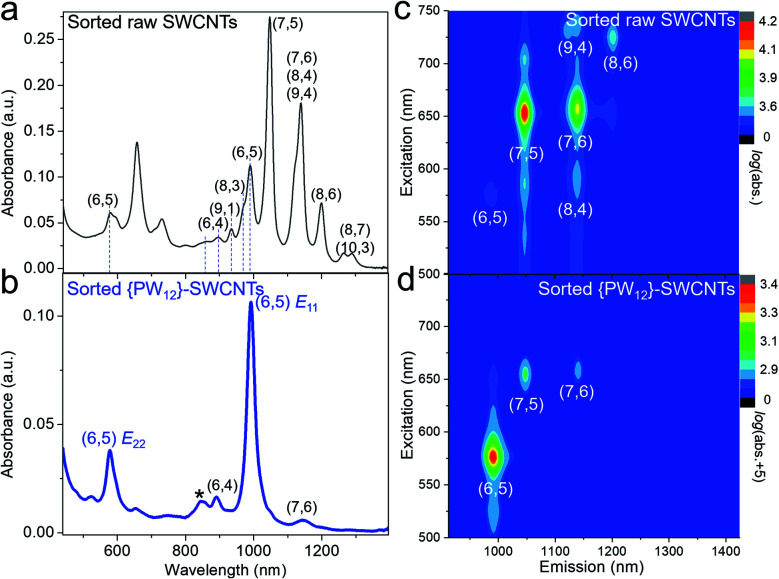
(a and b) UV-vis-NIR absorption spectra of PCz-sorted raw SWCNTs (a) and {PW_12_}-SWCNTs (b). The star at 861 nm marked in (b) indicates the phonon side-band of (6,5) SWCNTs. (c and d) PL maps of PCz-sorted raw SWCNTs (c) and {PW_12_}-SWCNTs (d).

Fig. 2 shows the UV-vis-NIR absorption spectra and PL maps of the PCz dispersed raw SWCNTs and {PW_12_}-SWCNTs under the same processing conditions. When sorting raw CoMo-SWCNTs by PCz, the dispersion consisted of an ensemble of SWCNTs with various chiralities, mainly including (7,5), (7,6), (6,5), and (8,6), and show some tendency to (7,5) (Fig. 2a and c). For the PCz-sorted {PW_12_}-SWCNT dispersion, we observed strong peaks (990 nm and 576 nm) and a weak peak (861 nm) attributable to the absorption of the van Hove optical transitions (*E*_11_, *E*_22_) and the phonon side-band of (6,5) SWCNTs,^33^ respectively, indicating the chirality selectivity (Fig. 2b). The Raman spectrum of sorted (6,5) SWCNTs did not exhibit an obvious defect-induced *D* band compared with that of raw CoMo-SWCNTs (Fig. S6†). We also performed PL characterization to further determine the chirality indices of the SWCNTs. As shown in Fig. 2d, the main fluorescence peak for the (6,5) species appears in PCz-sorted {PW_12_}-SWCNTs. The purity of the (6,5) SWCNTs was estimated to be 90.6% by the quantitative analysis of the absorption spectrum (Fig. S7 and Table S1†), which is close to the purity of (6,5) SWCNTs sorted by other reported methods.^35,37^ Both the absorption and PL characterization demonstrate that nearly monochiral (6,5) SWCNTs were extracted from {PW_12_}-SWCNTs by PCz wrapping.

### Sorting mechanism

We suggest that the selective separation of small-diameter (6,5) SWCNTs may arise from the steric hindrance effect of {PW_12_} clusters adsorbing on out surface of large-diameter SWCNTs, thereby preventing the polymer wrapping the large-diameter SWCNTs, as schematically shown in Fig. 1a.

**Fig. 3:**
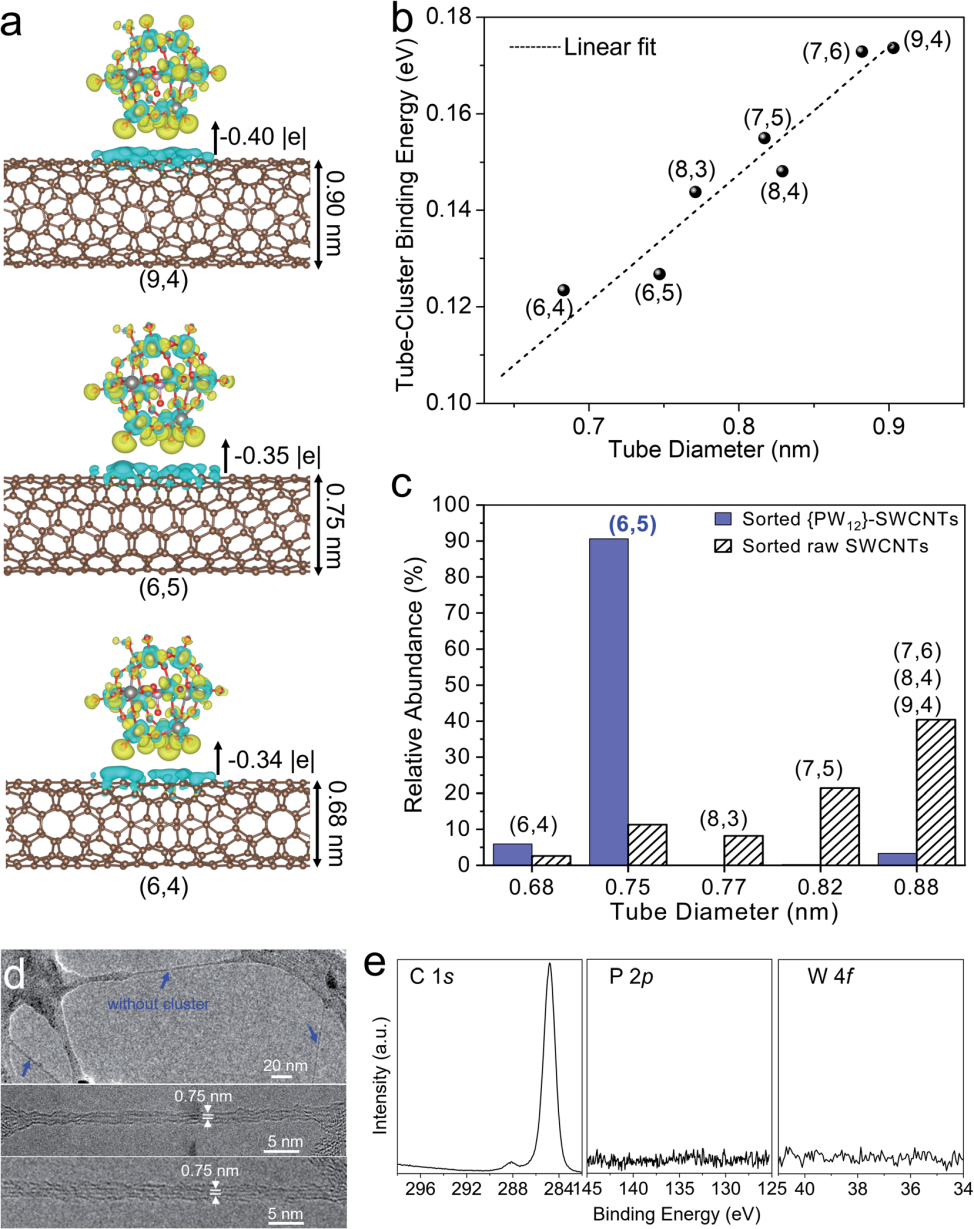
(a) DFT calculations: iso-surface plots of the electron density differences for {PW_12_} on the outside of SWCNTs with different chiralities/diameters: (6,4), (6,5), and (9,4). The corresponding electron transfer (|*e*|) is indicated. (b) Binding energy of (*n*,*m*) SWCNTs with {PW_12_}. The fitting line gives *y* = 0.26*x* – 0.06, with an *R*^2^ = 0.96, and shows a linear correlation between the tube diameter and binding energy. (c) Histogram showing the relative (*n*,*m*) abundance of PCz-sorted raw SWCNTs and {PW_12_}-SWCNTs based on the absorption spectra. (d) TEM images of sorted {PW_12_}-SWCNTs wrapped by the polymer. Blue arrows indicate the SWCNTs without clusters. (e) XPS spectra of the PCz-sorted {PW_12_}-SWCNT film.

In order to understand the chirality selective sorting of the SWCNTs, DFT calculations were carried out to unveil the interaction between the {PW_12_} clusters and various (*n*,*m*) SWCNTs. A series of (*n*,*m*) SWCNTs with different diameters that exist in raw CoMo-SWCNTs were adopted. The optimized configurations of {PW_12_}-SWCNTs have an inter-distance of around 3.36 Å between the SWCNT and charge neutral {PW_12_} (Fig. S8†), indicating the weak van der Waals interaction between the SWCNTs and outside {PW_12_} clusters. The electron transfer from the nanotube to {PW_12_} associated with the {PW_12_}–SWCNT interaction was revealed through DFT calculations along with Bader charge analysis, and generally increases with increased tube diameter (Fig. 3a and S9†). Additionally, we found that the binding energy of {PW_12_}–SWCNT is positively proportional to the tube diameter (Fig. 3b). A possible reason is that the convex SWCNT area in contact with the clusters increases with decreased tube curvature (*i.e.*, increased tube diameter). Therefore, during sonication, the weak association of the {PW_12_} cluster leads to preferential loss of the clusters from the small-diameter SWCNT surface, thereby enabling the polymer to selectively wrap and isolate these first released small-diameter SWCNTs. In contrast, for large-diameter SWCNTs exhibiting stronger interactions with {PW_12_}, the clusters act as steric hindrance on the outer surface of the nanotubes to block polymer wrapping (Fig. 1a).

The DFT calculations are consistent with the experimental results. We compared the relative chirality abundance of the PCz-sorted {PW_12_}-SWCNTs based on deconvoluting the absorption spectra with those of the sorted raw SWCNTs (Fig. S7, Tables S1 and S2†). It was found that small-diameter (6,4) and (6,5) tubes sorted from {PW_12_}-SWCNTs are more abundant than those sorted from raw SWCNTs (Fig. 3c), which is consistent with DFT calculations showing that these small-diameter (6,4) and (6,5) tubes exhibit weaker interactions with the clusters than the large-diameter SWCNTs (*e.g.*, (8,3), (7,5), (7,4), *etc.*). As (6,4) species are very few in raw CoMo-SWCNTs (Fig. S5†), therefore the (6,5) SWCNTs are highly enriched in the dispersion. Additionally, as compared with (6,5) tubes sorted from raw SWCNTs, (6,5) tubes extracted from {PW_12_}-SWCNTs did not exhibit charge transfer induced spectral shifts (Fig. 2),^75^ implying that clusters detached from the (6,5) tubes after sorting. This was further demonstrated by both TEM and XPS at the micro and macroscale, respectively. Fig. 3d depicts the aberration-corrected TEM images of sorted SWCNTs wrapped by the polymer. The sorted nanotubes exhibit a diameter of 0.75 nm, which is close to that of the (6,5) SWCNTs. As would be expected for the steric hindrance effect-based selective separation, no clusters were observed on the sorted SWCNTs (Fig. 3d), whereas a large number of clusters remained in the precipitation of SWCNT bundles after centrifugation (Fig. S10†). The XPS of the sorted (6,5) SWCNT film recorded around the C 1s, P 2p, and W 4f core levels does not show signals of P or W, further affirming the absence of {PW_12_} on sorted (6,5) SWCNTs (Fig. 3e).

### Universality of separation with various polyoxometalates

**Fig. 4:**
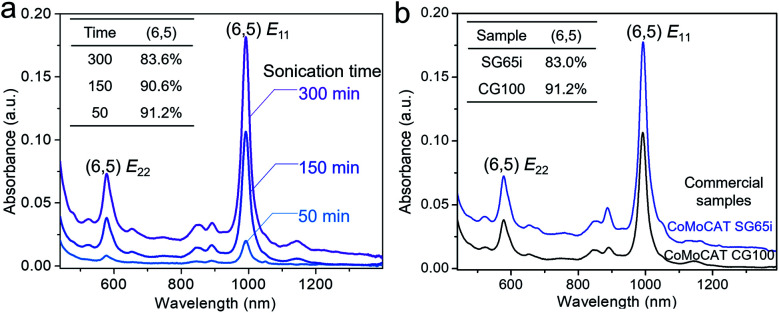
(a) Absorption spectra of PCz sorted {PW_12_}-SWCNTs under different sonication times: 50, 150, and 300 min. Inset: normalized spectra. Other conditions are the same. (b) Absorption spectra of PCz-sorted {PW_12_}-SWCNTs from two commercial samples: CoMoCAT SG65i and CoMo CG100, in which (6,5) chirality is more enriched in the former one.

Inspired by the sorting mechanism associated with the cluster steric hindrance, we improved the sorting efficiency by enhancing the sonication time. When prolonging the sonication time from 50 min to 300 min, the absorbance of (6,5) SWCNTs was improved by 9 times and the (6,5) chirality selectivity remained similar (91.2–90.6%) at 50–150 min and slightly decreased at 300 min (83.6%) when comparing the normalized absorption spectra (Fig. 4a, inset, Fig. S11†). This result indicates that the concentration of sorted (6,5) SWCNTs increases with the prolonged sonication time, because the prolonged sonication causes the detachment of more clusters from small-diameter (6,5) nanotubes and, resultantly, more released (6,5) SWCNTs were extracted by polymers. It was found that the defect induced *D* band did not obviously change between these samples (Fig. S12†), indicating that the prolonged sonication (300 min) did not cause further damage to the nanotube structure. We also carried out scanning electron microscopy (SEM) to measure the tube length. The average length of the (6,5) SWCNTs sonicated for 300 min was determined to be 1.75 ± 0.71 μm (Fig. S13†), which is similar to that of the (6,5) tubes separated by the shear force mixer method^55^ but is longer than that of the semiconducting tubes by polymer extraction.^47^ We also used another commercialized CoMoCAT SWCNT (SG65i) enriched with (6,5) chirality (∼41%) to assemble with {PW_12_} clusters and performed the separation. The sorted (6,5) SWCNTs by PCz with the same conditions show a significantly stronger absorbance of (6,5) species than those extracted from nonchiral-selective CoMoCAT SWCNTs (CG100) (Fig. 4b). This result again proved that the sorting strategy is favorable for selecting (6,5) SWCNTs.

**Fig. 5:**
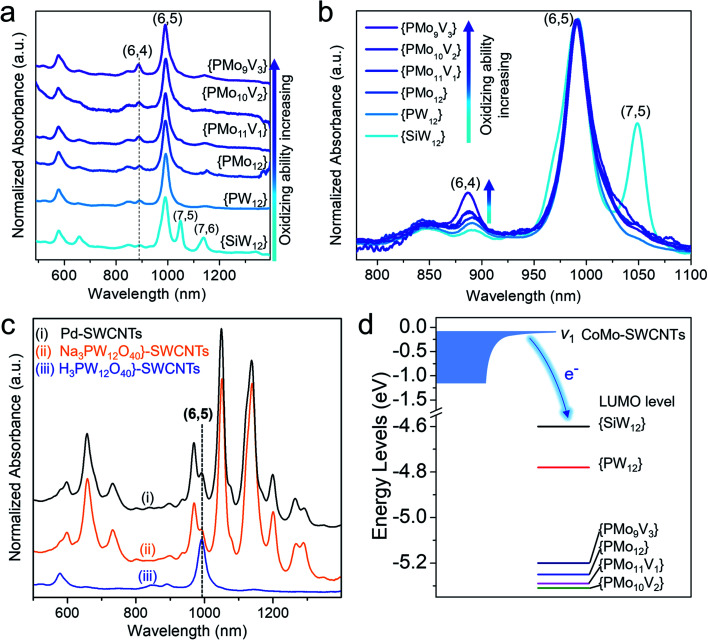
(a and b) Absorption spectra of PCz-sorted SWCNTs assembled with {SiW_12_}, {PW_12_}, and {PMo_12−*x*_V_*x*_} clusters. The spectra were normalized with respect to the *E*_11_ peaks of the (6,5) SWCNTs (a) and corresponding stacking spectra (b). (c) Comparison of the absorption spectra of PCz-sorted SWCNTs assembled with the acid H_3_PW_12_O_40_, salt Na_3_PW_12_O_40_, and Pd nanoparticles. (d) Pictorial representation of the LUMO levels of polyoxometalates and the top of the valence band (*v*_1_) of CoMo-SWCNTs.

This sorting strategy was further expanded to other redox-active polyoxometalates. A series of Keggin type polyoxometalates with a similar size of ∼1 nm, whose oxidizing abilities are in the order^76^ H_3+*x*_PMo_12−*x*_V_*x*_O_40_ > H_3_PMo_12_O_40_ > H_3_PW_12_O_40_ > H_4_SiW_12_O_40_ (denoted as {PMo_12−*x*_V_*x*_} (*x* = 1, 2, 3), {PMo_12_}, {SiW_12_}), were used to assemble with CoMo-SWCNTs. The selective separation of (6,5) SWCNTs by polymer wrapping was proven to be valid in all these polyoxometalates except for {SiW_12_} (Fig. 5a). Because of the weakest oxidizing ability of {SiW_12_}, the differences in the interaction between {SiW_12_} and various (*n*,*m*) SWCNTs decreased. Therefore, sonication drives the {SiW_12_} detachment from diverse SWCNTs, thereby resulting in the non-chirality selectivity toward the sorted SWCNTs. Similar result of the poor chiral selectivity of SWCNTs was also obtained when using a sodium salt (Na_3_PW_12_O_40_) with a weaker oxidizing ability than its acid (H_3_PW_12_O_40_) (Fig. 5c(ii)). For comparison, we also prepared ∼1–2 nm Pd nanoparticles without oxidizing ability (Fig. S14†) and performed the separation of Pd-SWCNT assemblies under the same conditions. This also shows that the weak interaction (*i.e.*, no electron transfer) of Pd-SWCNTs leads to the non-chirality selectivity (Fig. 5c(i)). Additionally, when normalizing the spectra with respect to the *E*_11_ peaks of the (6,5) SWCNTs, it is found from stacking the spectra that the stronger the oxidizing ability of the polyoxometalates, the higher the content of smaller-diameter (6,4) SWCNTs (Fig. 5b, marked by an arrow). These experiments again demonstrate that the separation of chirality specific SWCNTs depends not only on the cluster steric hindrance effect but also on the oxidizing ability of the polyoxometalates.

To understand the effect of the polyoxometalate’s oxidizing ability on sorting the SWCNTs, we measured the relative positions of the LUMO levels (*i.e.*, redox potentials) of various polyoxometalates and the SWCNT valence band through cyclic voltammetry (Fig. 5d, S15 and S16†). It has been reported that the heteropolyacid LUMO energy is significantly lower than the top of the valence band of the SWCNTs, allowing the reduction potential of the clusters to be correlated directly to its encapsulation *via* the oxidation of the large-diameter (1–2 nm) nanotubes.^72^ We found that the top of the valence band of subnanometer CoMo-SWCNTs (*v*_1_, –0.08 eV) is higher than the LUMO levels of all polyoxometalates (−4.6 to −5.3 eV) (Fig. 5d, S15 and S16†). This permits spontaneous charge transfer from the SWCNTs to the relative lower lying LUMOs of the polyoxometalates, facilitating the interaction of the polyoxometalates with the SWCNTs. The larger the gap between the LUMO levels of the polyoxometalate and the valence band of the SWCNTs, the stronger the interaction between the polyoxometalate and nanotube is, which is favorable for the steric hindrance induced sorting. Therefore, the critical LUMO level of polyoxometalates for sorting (6,5) SWCNT is between −4.6 eV ({SiW_12_}) to −4.8 eV ({PW_12_}) (Fig. 5d). This diagram provides a clear design rule for the sorting of chirality specific SWCNTs based on the precise matching of the redox potentials of the SWCNTs and polyoxometalates.

## Conclusion

We developed a cluster steric hindrance-based strategy by assembling polyoxometalates on the outside of CoMo-SWCNTs and demonstrated the extraction of (6,5) SWCNTs with a diameter of 0.75 nm by the conjugated polymer PCz. Aided by DFT calculations, TEM, and XPS characterizations, we rationalized that the selective separation was associated with the tube diameter-dependent interaction between the tubes and polyoxometalates. During sonication, the polyoxometalates preferentially detached from the small-diameter (6,5) SWCNTs with weak interactions, thereby enabling the polymer to selectively wrap and separate the released (6,5) SWCNTs, while strong binding clusters with the large-diameter SWCNTs acted as steric hindrance and blocked the polymer wrapping. Based on this strategy, the sorting efficiency of (6,5) SWCNTs was improved by prolonging the sonication time, with clusters continuously detaching from (6,5) SWCNTs. The universality of a series of polyoxometalates demonstrated that the separation of chirality specific SWCNTs depends not only on the cluster steric hindrance effect but also on the oxidizing ability of the polyoxometalates. This mechanism is different from other sorting methods and has not been reported previously.

We believe that the great feasibility and robustness of this strategy by using polyoxometalates to precisely manipulate the electronic structures of SWCNTs can be extended to achieve the sorting of more single-chirality SWCNTs. We expect the combination of the multiscale structure of polyoxometalates and low-dimensional materials will expand their applications in a variety of fields, including separation and nanofabrication.

## Experimental section

### Preparation of cluster-SWCNTs

The {PW_12_} and {SiW_12_} were purchased from Meryer (Shanghai) Chemical Technology Co., Ltd., the {PMo_12_} were purchased from Shanghai Xianding Biological Technology Co., Ltd, and raw CoMo-SWCNTs (CG100, tube purity > 89%) were purchased from Sigma Aldrich. The {PMo_12−*x*_V_*x*_} (*x* = 1, 2, 3) were synthesized by adopting a literature procedure.^77^

Typically, 40 mg of CoMo-SWCNTs were added into aqueous {PW_12_} solution (800 mg {PW_12_}/12 mL H_2_O) and stirred for 48 h at room temperature. Then, through centrifuging and washing the precipitation 10 times with deionized water, the excess aggregated {PW_12_} clusters on the outside of the nanotubes were removed. Finally, the {PW_12_}-SWCNTs hybrids were dried in an oven at 80 °C. Other polyoxometalates were assembled with SWCNTs by using the same method. It is worth noting that a plastic spoon should be used for sampling due to the oxidation of the heteropolyacid by a metal spoon.

### Separation of the SWCNTs

Commercial PCz ((C_29_H_41_N)_*n*_, *M*_w_ > 50 000 g mol^−1^) was purchased from Mreda Technology Co., Ltd., China. Typically, 10 mg of PCz was dissolved in 20 mL of toluene and then mixed with 5 mg of the {PW12}-SWCNTs. The mixture was then sonicated using a tip ultrasonic disintegrator (Scientz-IID, Ningbo Scientz Biotechnology Co., Ltd., China) at 500 W in the pulse mode and 2 s on/3 s off intervals for 50–300 min. The mixture was cooled in an ice water bath (5 °C) during sonication. The obtained dispersion was centrifuged at 13 000 g for 10 min, then the supernatant (upper 80%) was collected for further measurements.

### Characterization of the SWCNTs

HAADF-STEM was carried out on an FEI Titan Cubed Themis G^2^ 300 apparatus with an X-FEG electron gun and a DCOR aberration corrector operating at 300 kV. TEM was performed on an aberration corrected ETEM FEI Titan G^2^ 80–300 operating at 300 kV. SEM was performed on a ZEISS Merlin SEM operating at 1.0 kV. UV-vis-NIR absorption spectra were collected using a Shimadzu (UV-2600). PL was measured using a spectrofluorometer (Nanolog, HORIBA) equipped with a liquid nitrogen-cooled InGaAs near-infrared array detector. The excitation wavelength was varied from 500 to 750 nm in 5 nm steps, and the emission wavelength was varied from 900 nm to 1450 nm in 5 nm steps. XPS spectroscopy was conducted on a ThermoFisher™ ESCALAB™ Xi + photoelectron spectrometer equipped with a monochromatic Al Kα X-ray source. Analyses were performed on an area with a diameter of 500 μm. All peaks were calibrated with the C 1s peak binding energy at 284.8 eV.

### Computational details

The DFT calculations were performed using the Vienna *ab initio* simulation package software. The structural relaxation employed the generalized gradient approximation (GGA) formulated by Perdew, Burke and Ernzerhof (PBE). The projector augmented wave potential (PAW) method was used in the calculations. A kinetic cut-off energy of 400 eV for the plane wave basis set was chosen and a 1 × 1 × 1 *k*-point mesh was generated. All the ionic relaxations were optimized by the recommended conjugate gradient algorithm until the maximum atomic force component acting on each atom was less than 0.01 eV Å^−1^.

We designed the {PW_12_}-SWCNT model, *i.e.*, a charge neutral {PW_12_} cluster residing around the SWCNT. Periodic boundary conditions were considered with the axial direction of the nanotube along the *c* axis. The cell parameters for *a* and *b* were set to be 35 Å to prevent the interference of the periphery images. A series of (*n*,*m*) SWCNTs with different diameters were used to reveal the interactions of the {PW_12_}-SWCNTs. The charge density difference was calculated to present the charge transfer between {PW_12_} and SWCNT. The binding energy (*E*_B_) is given by*E*_B_ = *E*_SWCNT_ + *E*_PW12_ − *E*_PW12–SWCNT_

### Cyclic voltammetry measurement

Cyclic voltammetry of the different polyoxometalates was performed in Na_2_SO_4_ solution (0.5 mol L^−3^) using a 3 mm diameter glass carbon working electrode, a carbon rod counter electrode, and a saturated calomel reference electrode. The LUMO levels of the polyoxometalates were calculated by the following method:^78^*E*_LUMO_ (eV) = −e(4.5 + 0.241 + *E*_red_)where *E*_red_ was determined from the cyclic voltammogram (Fig. S15†). For the cyclic voltammetry testing of the SWCNTs, CoMo-SWCNTs (8 mg) and 1 mL DMF were first sonicated in a bath for 1 h. Then, the resulting suspension (8 μL) was dropped onto glassy carbon as the working electrode and allowed to dry. The voltammetry of CoMo-SWCNTs was performed with a 3 mm glassy carbon working electrode, a carbon rod counter electrode, and a Ag/Ag^+^ reference electrode in 0.5 mol L^−3^ Na_2_SO_4_ solution. To fairly compare with the voltammogram of polyoxometalates obtained with the calomel reference electrode, we converted the potential (V *vs.* Ag/Ag^+^) of the voltammogram of the SWCNTs to the potential (V *vs.* SCE) by subtracting 45 mV (Fig. S16†).

## Data availability

All data have been in the main text and ESI.†

## Author contributions

F. Y. contributed to the idea and experimental design and wrote the manuscript. X. Y., C. Z., W. X., Luyao Zhang, Lei Zhang, and K. Z. prepared the samples, collected the data, and analyzed the results. Lianduan Zeng and X. W. contributed to the DFT calculation. M. L. and Y. L. provided the PL measurements and discussed the sorting mechanism. All authors contributed to data analysis, interpreted the data, and approved the final manuscript.

## Conflicts of interest

The authors declare the following competing financial interest(s): F. Y, X. Y., and Y. L. declare a financial interest: patents related to this research have been submitted. The remaining authors declare no competing financial interests.

## Supplementary Material

SC-013-D2SC01160C-s001
