# Supplementary material for: Polyoxometalate steric hindrance driven chirality-selective separation of subnanometer carbon nanotubes
Source: Chem Sci. 2022 Apr 25;13(20):5920–8. doi: 10.1039/d2sc01160c (PMC9132071; doi:10.1039/d2sc01160c)
Supplement: SC-013-D2SC01160C-s001 [file SC-013-D2SC01160C-s001.pdf]

Electronic Supplementary Material (ESI) for Chemical Science.
This journal is © The Royal Society of Chemistry 2022

Supporting Information

Polyoxometalate Steric Hindrance Driven Chirality-Selective Separation of Subnanometer Carbon Nanotubes

Xusheng Yang, Chao Zhu, Lianduan Zeng, Weiyang Xue, Luyao Zhang, Lei Zhang, Kaitong Zhao, Min Lyu, Lei Wang, Yuan-Zhu Zhang, Xiao Wang*, Yan Li*, and Feng Yang*

Supporting Results

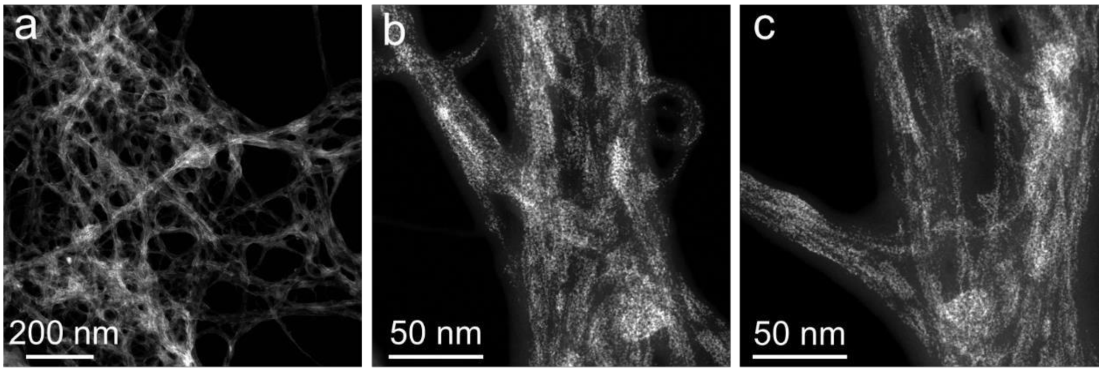

Figure S1. **a**–**c**, Typical HAADF-STEM images of {PW_{12}}-CoMo-SWCNTs.

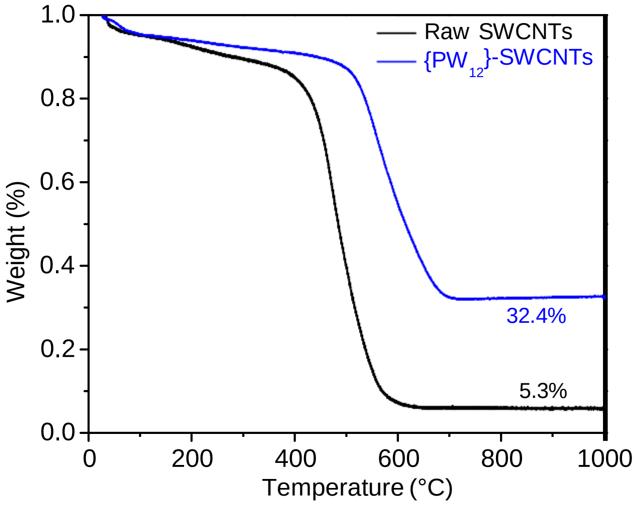

Figure S2. Thermogravimetric curves of {PW_{12}}-SWCNTs and raw SWCNTs.

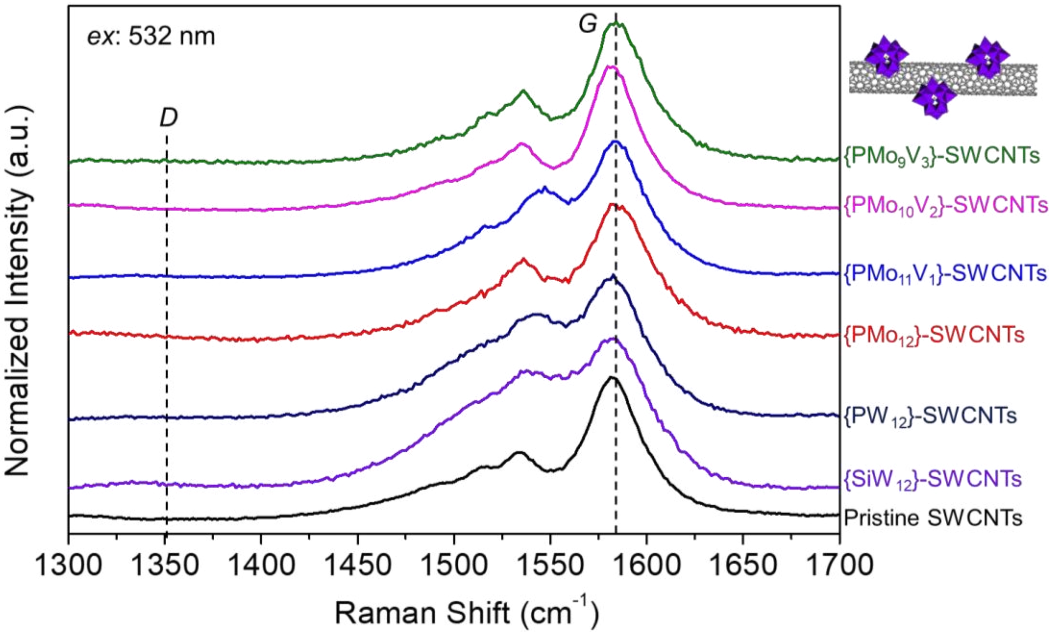

Figure S3. Raman spectra of polyoxometalate-SWCNTs and pristine SWCNTs. The spectra were normalized with respect to the intensity of *G* bands. A laser filter (25%) was applied to the powder sample to avoid any laser heating effect induced Raman shift.

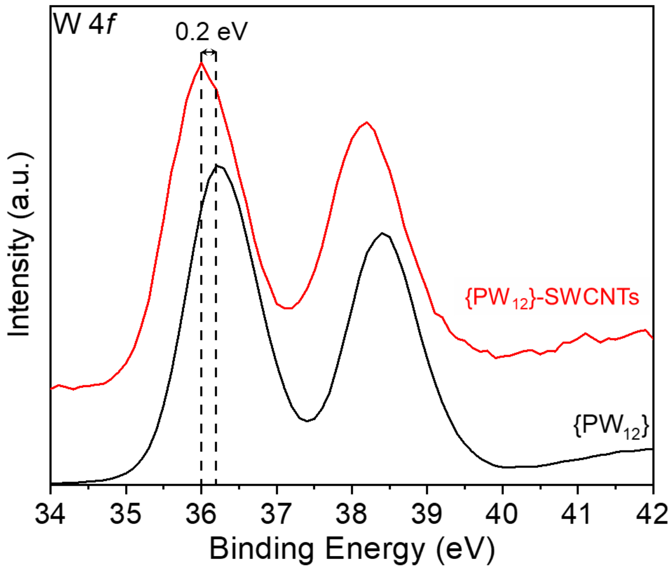

Figure S4. XPS spectra of $\{PW_{12}\}$ and $\{PW_{12}\}$-SWCNTs.

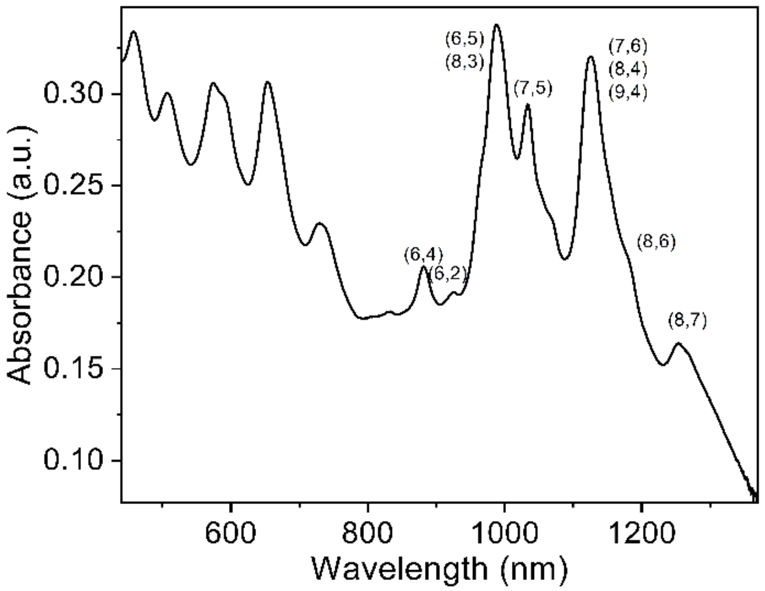

Figure S5. Absorption spectra of sodium deoxycholate dispersed raw CoMo-SWCNTs, indicating the non-selective chiral indices in raw sample.

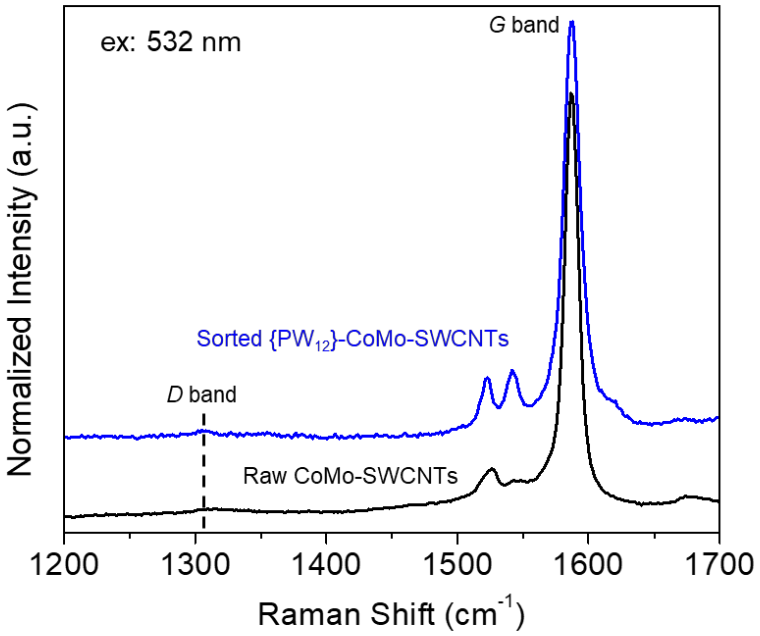

Figure S6. Raman spectra of sorted $\{PW_{12}\}$-CoMo-SWCNTs and raw CoMo-SWCNTs. The spectra are normalized with the *G* bands.

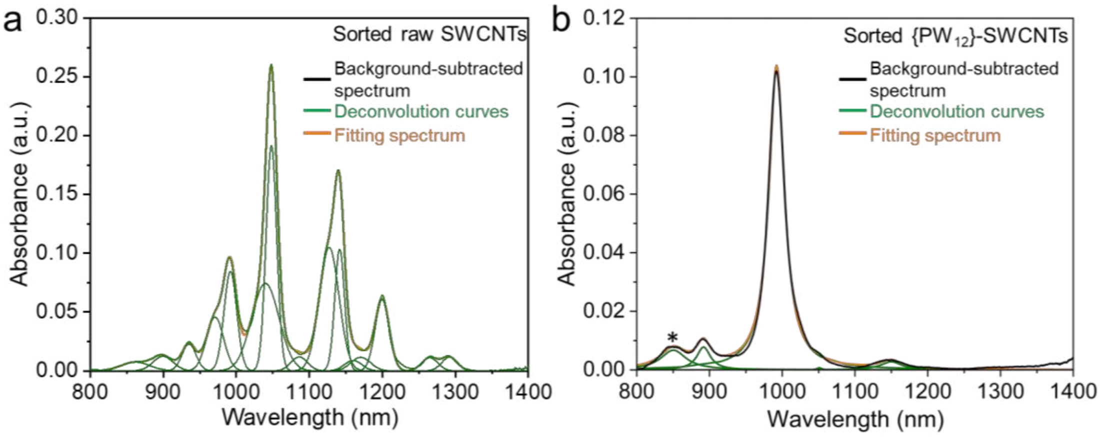

Figure S7. UV–Vis–NIR absorption spectra, deconvolution curves, and fitting spectra of PCz sorted raw SWCNTs (**a**) and {PW_{12}}-SWCNTs (**b**).

Table S1. Fitted and calculated relative abundance of (*n*,*m*) in PCz sorted {PW_{12}}-SWCNTs from absorption spectra.

(*n*,*m*)	Wavelength (nm)	Peak Area	Relative Abundance (%)
(6,4)	891	0.27	5.9
(6,5)	992	4.14	90.6
(7,5)	1028	0.01	0.2
(7,6)	1146	0.15	3.3

Table S2. Fitted and calculated relative abundance of (*n*,*m*) in PCz sorted raw SWCNTs from absorption spectra.

(*n*,*m*)	Wavelength (nm)	Peak Area	Relative Abundance (%)
(6,4)	899	0.42	2.7
(9,1)	935	0.55	3.6
(8,3)	970	1.34	8.8
(6,5)	992	1.85	12.1
(7,5)	1048	3.49	22.9
(7,6)(8,4)(9,4)	1125-1160	5.54	36.3
(8,6)	1200	1.47	9.6
(8,7)	1265	0.30	2.0
(10,3)	1292	0.31	2.0

Three types of adsorption sites of {PW_{12}} on a (8,4) SWCNT were used. The smallest $O_{cluster}$-C_{CNT} inter-distance was found to be 1.44, 2.44, and 3.41 Å (**Figure

S8a–**c**), respectively. The optimized configuration by DFT calculations is determined to be 3.40 Å (**Figure S8d**).

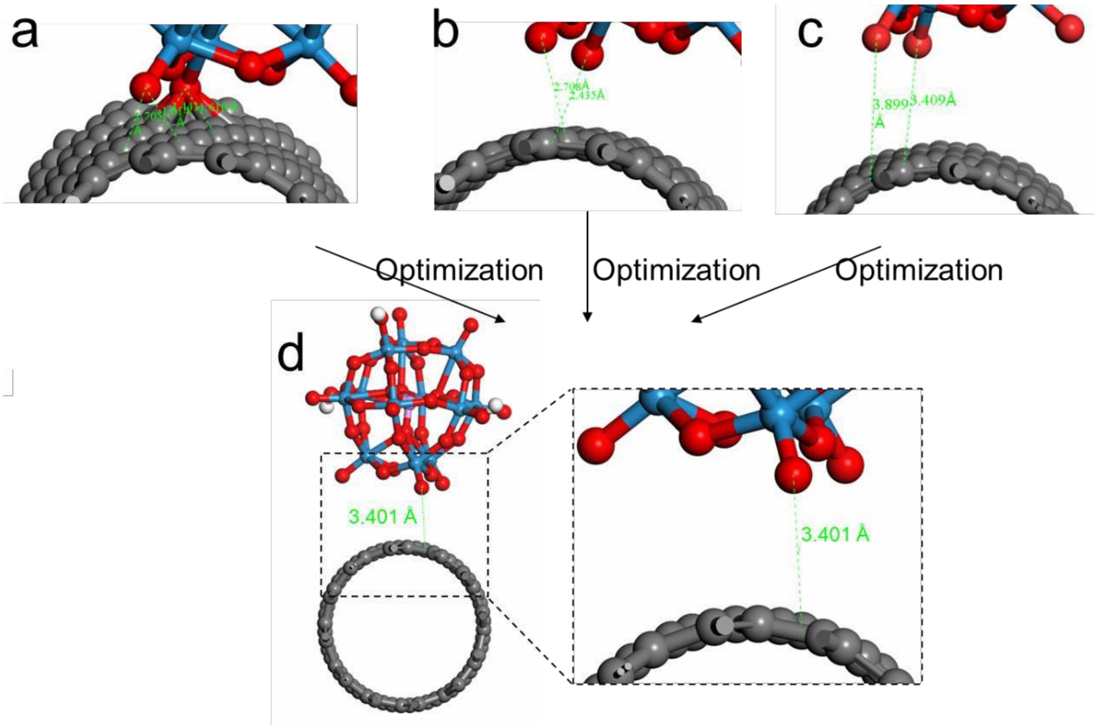

Figure S8. **a**–**c**, Different adsorption sites of {PW_{12}} on (8,4) SWCNT before optimization. The smallest inter-distances (O-C) 1.44 (**a**), 2.44 (**b**), and 3.41 Å (**c**) are labelled. **d**, Optimized configuration of {PW_{12}}/(8,4) SWCNT in tri-adsorption site with an inter-distance of 3.40 Å.

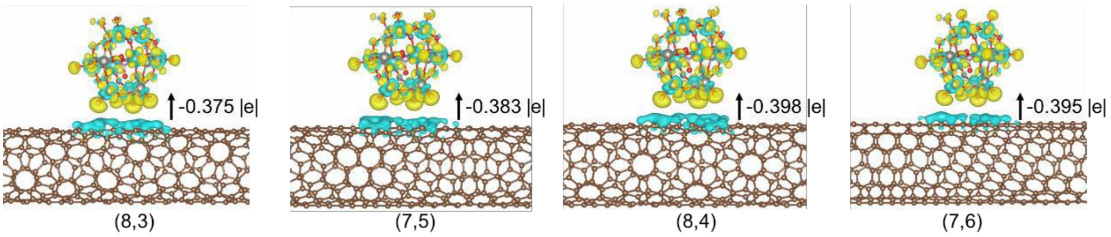

Figure S9. DFT calculations: iso-surface plots of electron density differences for {PW_{12}} on outside SWCNTs with different chirality/diameter: (8,3), (7,5), (8,4), and (7,6). The corresponding electron transfer (|e|) is indicated.

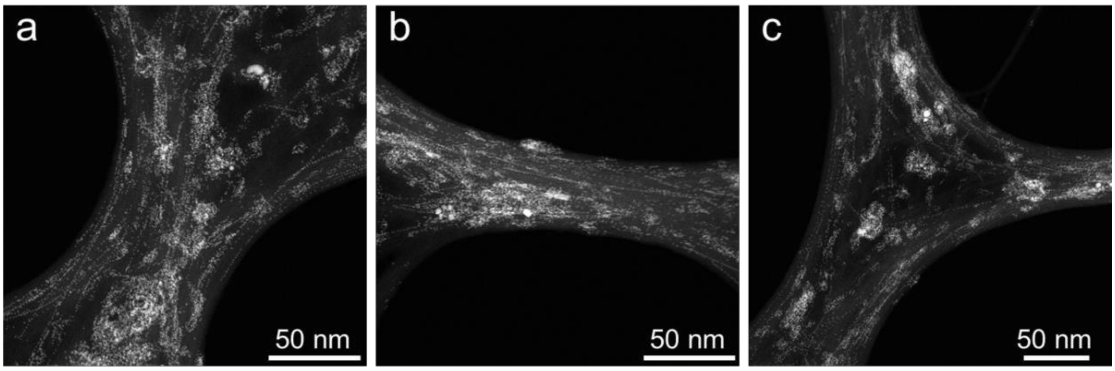

Figure S10. **a**–**c**, HAADF-STEM images of $\{PW_{12}\}$-SWCNT residue safter the sorting and centrifugation, showing lots of $\{PW_{12}\}$ clusters adsorbed on outside SWCNTs.

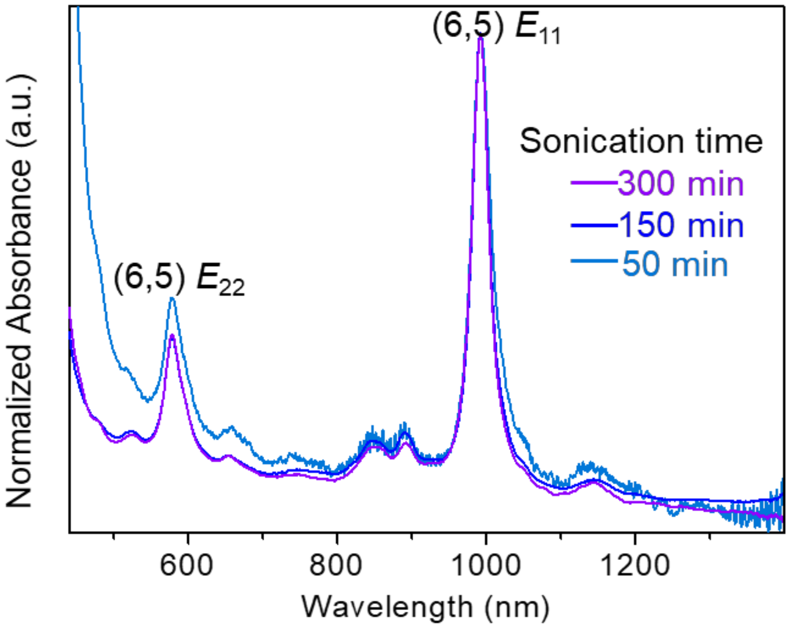

Figure S11. Normalized absorption spectra of PCz sorted $\{PW_{12}\}$-SWCNTs under different sonication time: 50, 150, and 300 min.

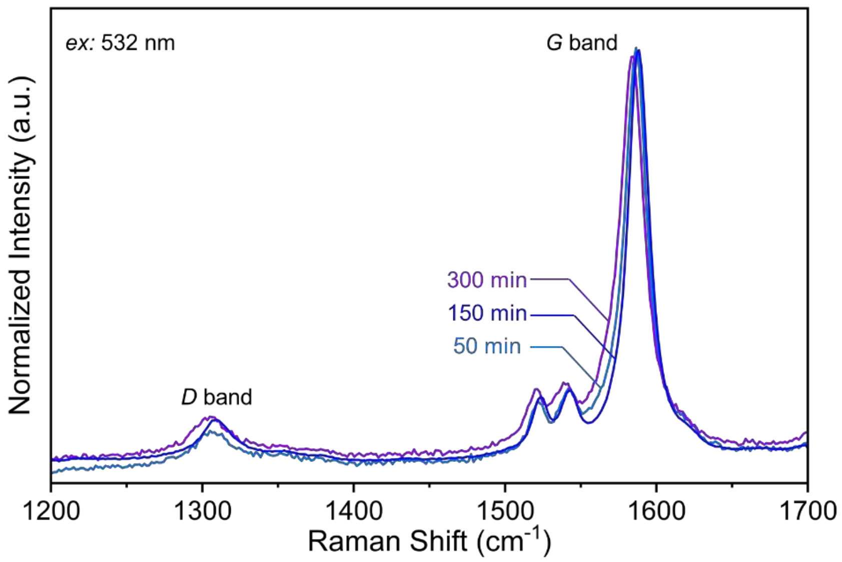

Figure S12. Raman spectrum showing the *D*, *G* bands of sorted SWCNTs via different sonication time.

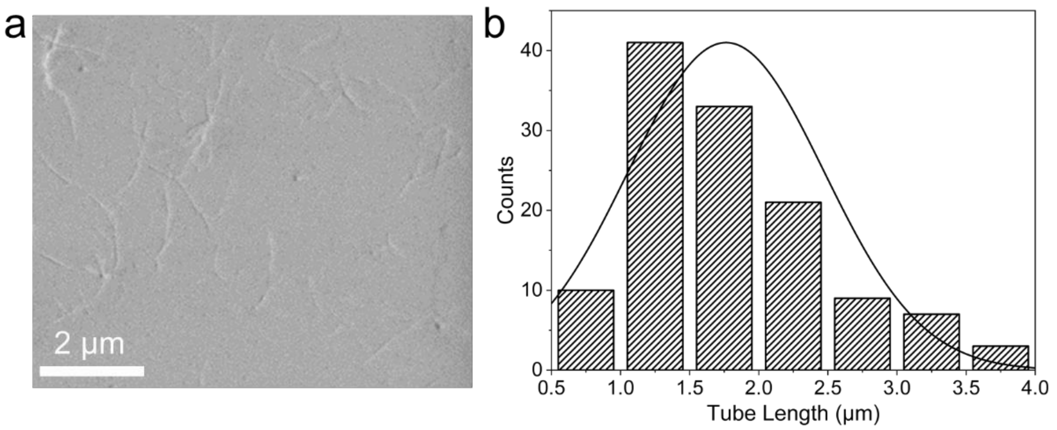

Figure S13. SEM image (**a**) and length distribution (**b**) of (6,5) SWCNTs sonicated by 300 min. Tube counts: *N* = 124. Average length: 1.75 ± 0.71 μm.

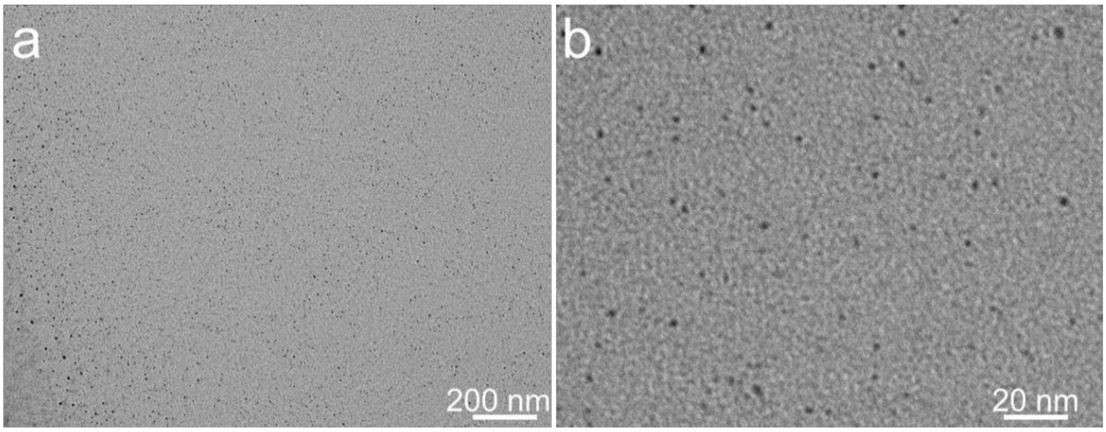

Figure S14. **a**, **b**, TEM images of Pd nanoparticles with a size of ~1-2 nm.

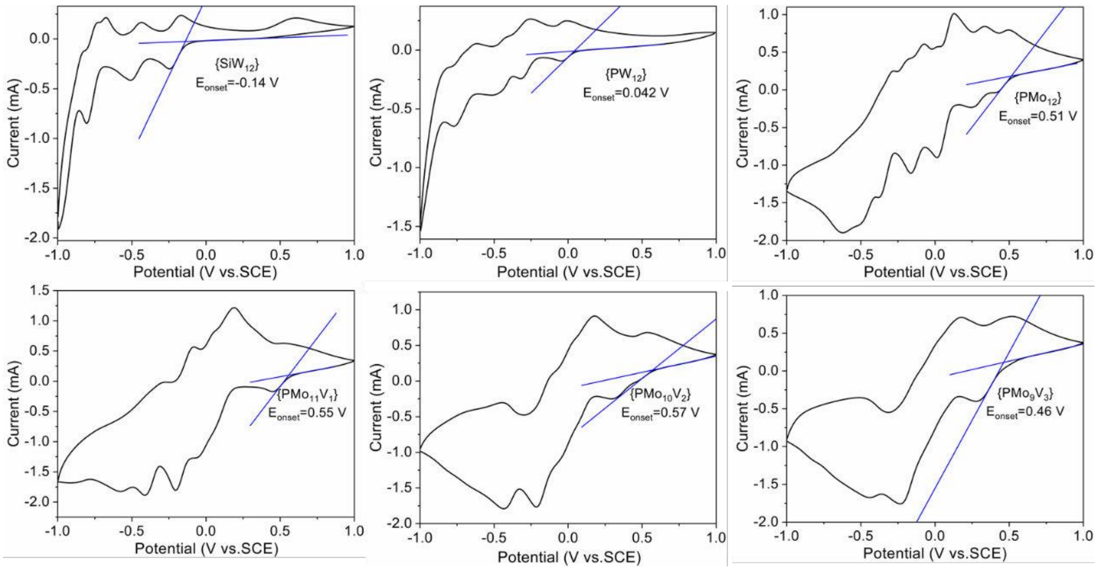

Figure S15. Cyclic voltammogram of different polyoxometalates in Na_2SO_4 solution (0.5 mol·L^{-3}) using a 3 mm-diameter glass carbon electrode, a carbon rod counter electrode, and a saturated calomel electrode (SCE) reference electrode.

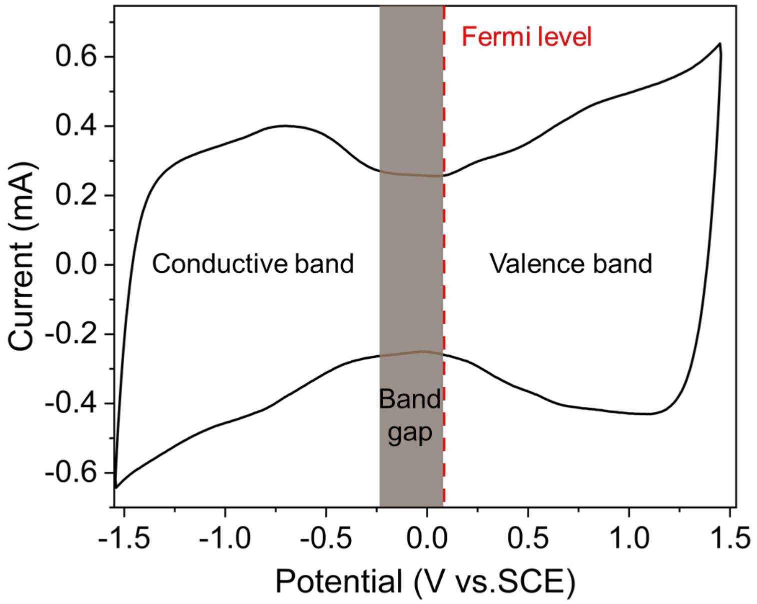

Figure S16. Cyclic voltammogram of CoMo-SWCNT thin film in Na_2SO_4 solution (0.5 mol·L^{-3}) using a 3 mm-diameter glass carbon electrode, a carbon rod counter electrode, and a Ag/Ag^{+} reference electrode. The potential (V vs. Ag/Ag+) of voltammogram of SWCNTs was converted to the potential (V vs. SCE) by subtracting 45 mV. The key features of the cyclic voltammogram are labeled.
